# A Novel XGBoost Method to Identify Cancer Tissue-of-Origin Based on Copy Number Variations

**DOI:** 10.3389/fgene.2020.585029

**Published:** 2020-11-20

**Authors:** Yulin Zhang, Tong Feng, Shudong Wang, Ruyi Dong, Jialiang Yang, Jionglong Su, Bo Wang

**Affiliations:** ^1^College of Mathematics and Systems Science, Shandong University of Science and Technology, Qingdao, China; ^2^College of Computer and Communication Engineering, China University of Petroleum (East China), Qingdao, China; ^3^Geneis (Beijing) Co., Ltd., Beijing, China; ^4^School of AI and Advanced Computing, XJTLU Entrepreneur College (Taicang), Xi’an Jiaotong-Liverpool University, Suzhou, China

**Keywords:** tissue-of-origin, copy number variations, multiclass, XGBoost, extremely randomized tree, principal component analysis

## Abstract

The discovery of cancer of unknown primary (CUP) is of great significance in designing more effective treatments and improving the diagnostic efficiency in cancer patients. In the study, we develop an appropriate machine learning model for tracing the tissue of origin of CUP with high accuracy after feature engineering and model evaluation. Based on a copy number variation data consisting of 4,566 training cases and 1,262 independent validation cases, an XGBoost classifier is applied to 10 types of cancer. Extremely randomized tree (Extra tree) is used for dimension reduction so that fewer variables replace the original high-dimensional variables. Features with top 300 weights are selected and principal component analysis is applied to eliminate noise. We find that XGBoost classifier achieves the highest overall accuracy of 0.8913 in the 10-fold cross-validation for training samples and 0.7421 on independent validation datasets for predicting tumor tissue of origin. Furthermore, by contrasting various performance indices, such as precision and recall rate, the experimental results show that XGBoost classifier significantly improves the classification performance of various tumors with less prediction error, as compared to other classifiers, such as K-nearest neighbors (KNN), Bayes, support vector machine (SVM), and Adaboost. Our method can infer tissue of origin for the 10 cancer types with acceptable accuracy in both cross-validation and independent validation data. It may be used as an auxiliary diagnostic method to determine the actual clinicopathological status of specific cancer.

## Introduction

Recent advances in molecular biology, e.g., genomics, proteomics, and metabolics, have resulted in a more accurate and specific prediction of tumor response to treatment, as well as trends in metastasis recurrence and prognosis. However, traditional detection methods, e.g., clinical, impact, and pathological examination, can only determine 50–80% of patients of metastasis cancer, while the remaining 20–50% of patients still cannot be determined (Chen et al., 2017) as more effective methods of diagnosis are required. In the metastasis of cancer, tumor cells are carried from the primary site to lymphatics, blood vessels, or other sites to continue to grow and form the same type of tumor. Biochemical biopsy of micrometastasis may lead to partial diagnosis and chaos due to the instability of biochemical indicators. Identifying the type and origin of cancer is important to determine the most appropriate treatment for cancer patients. In practice, errors in the uncertainties will become bigger, resulting in the error of diagnosis. The molecular expression profile of tumor cells in the metastatic focus is more similar to that in the primary site but different from that in the metastatic site, suggesting that we can trace the tumor origin according to the molecular expression profile of tumor cells in the metastatic site.

Many studies have attempted to use cancer biomarkers to predict the locations of primary tumors in CUP such as gene expression, miRNA and DNA methylation (Talantov et al., 2006; Clavell et al., 2008; Staub et al., 2010; Søkilde et al., 2014; Tang et al., 2017; Grewal et al., 2019). Gene expression patterns in tumors were the most widely used biomarkers for tumor classification and have achieved higher accuracy with machine learning algorithms. For instance, the support vector machines (SVMs) have been utilized as a multiclass classifier for the expression levels of 16,063 genes, achieving 78% classification accuracy (Ramaswamy et al., 2001). Seventy-nine optimal gene markers were selected and subsequently used as features for training of SVM classifier, achieving 89% classification accuracy with 13 classes (Tothill et al., 2005). SVM-RFE approach has been applied to select 154 top genes and classified the 22 common tumor types on the pan-cancer transcriptome database, obtaining an overall accuracy of 96.5% for training set and 97.1% accuracy for independent test set consisting of 9,626 primary tumors (Xu et al., 2016). Other machine learning classifiers have been also carried out to identify tissues of origin. For example, K-nearest neighbors (KNN) classifier has been used for 39 cancers including 92 genes, achieving 84% accuracy in cross-validation and 82% in the independent set of 112 samples (Ma et al., 2006). Neural networks have been applied to complementary DNA (cDNA) and oligonucleotide data consisting of training sets and test sets independently, achieving a mean accuracy of 83% (Bloom et al., 2004). Random forest classifiers have been utilized on publicly available somatic mutation data in the COSMIC database to train using leave-one-out cross-validation and achieved over 80% accuracy (Marquard et al., 2016). Random forest has been also used to establish classifiers for 38 kinds of tumors to methylation data and obtained 87% accuracy in the test set. A least absolute shrinkage and selection operator has been proposed using cross-validated algorithm, which achieved an overall accuracy of 85% (Søkilde et al., 2014). The artificial bee colony (ABC) and the particle swarm optimization have been carried out on the brain lower grade glioma data. The highest classification accuracy was 99.1% by the ABC algorithm (Bhowmick et al., 2019). Although these methods have achieved promising results, they had the disadvantages of lower classification accuracy on independent sets with more features.

Copy number variations (CNVs) usually refer to the individual difference in continuous DNA fragments with length ranging from several thousand to several trillion base pairs in genome. It can affect the individual phenotype by changing the number of gene copies, thus affecting the expression dose, disrupting the coding region structure of gene, changing the position or length of gene regulatory sequence, exposing recessive mutation, etc. (Redon et al., 2006; Poduri et al., 2013). So far, few studies have explored the effects of copy number variations on tissue-of-origin by machine learning algorithms. Zhang et al. (2015) have executed a comprehensive genome-wide analysis of 23,082 CNVs in 3,480 cancer patients, with six cancer types executed and 19 discriminative genes for tumor classification selected by minimum redundancy and minimum relevancy (mRMR) as well as incremental feature selection (IFS) methods. The overall prediction accuracy was about 75% in 10-fold cross-validation (Zhang et al., 2015).

**FIGURE 1:**
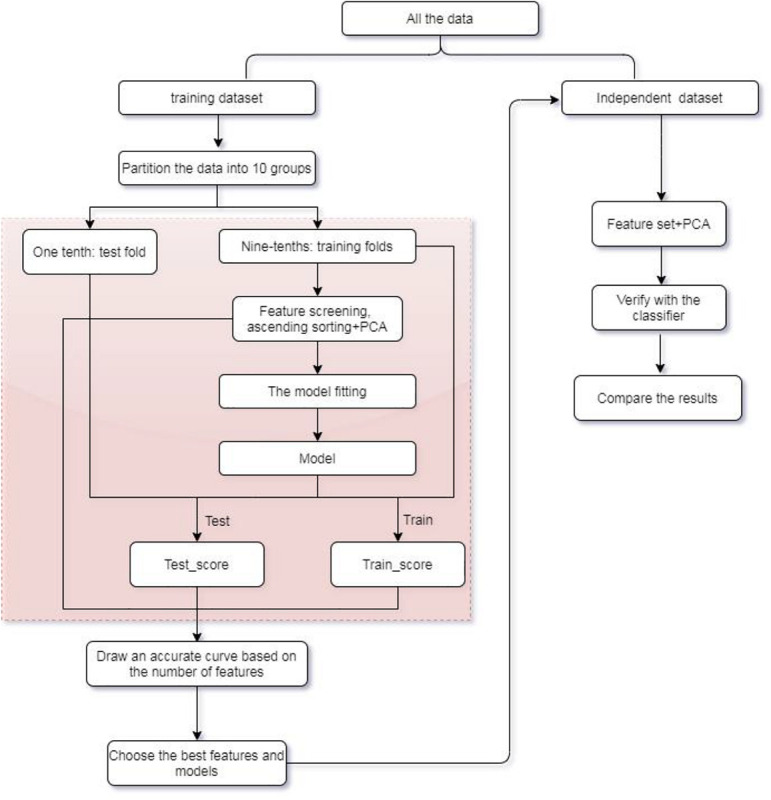
Flow chart of the method.

In this research, an efficient multiclass model of XGBoost is established to assist in the identification of tumor origin for the entire data group from 10 types of tumors based on copy number variations. Extremely randomized tree (Extra tree) is used to design an algorithm to select fewer variables replacing the original high-dimensional variables for training. Principal component analysis (PCA) is subsequently applied to feature extraction for eliminating noise. Our method is compared to algorithms, such as SVM, KNN, Bayes, and Adaboost in terms of its multiclass classification performance. Experimental results indicated that XGBoost classifier can achieve an overall higher accuracy of more than 0.8913 for all training dataset and 0.7421 for independent dataset with 300 features, significantly improving the classification performance of other classifiers. Refer to Figure 1 for the flow chart of our method.

This paper is organized as follows. A detailed description of CNV datasets are given first, then feature selection method and XGBoost classifier are introduced in section “Materials and Methods.” In section “Result and Discussion,” five classifiers including SVM, KNN, Bayes, Adaboost, and XGBoost are used to assess the prediction performance. The conclusions and discussion of the work are given in section “Conclusion.”

## Materials and Methods

### Data Preparation

The study focuses on the copy number value of 10 tumor datasets extracted from The Cancer Genome Atlas (TCGA). The copy number signal is produced by Affymetrix SNP 6.0 arrays for the set of samples in each TCGA study. These datasets are from primary solid tumor samples released by MSKCC in 2013, which are available from TCGA. The original data consist of 9,743 samples in total^1^. Each sample has 24,174 genes with discrete copy number value denoted as “−2,” “−1,” “0,” “1,” “2,” where “−2” is homozygous deletion, “−1” is heterozygous loss, “0” is diploid, “1” is one copy gain, and “2” is high-level amplification or multiple-copy gain. To evaluate the performance of CNVs_origin, we perform the experiments on another copy number datasets, which can be downloaded from http://gdac.broadinstitute.org/ released by TCGA in 2016 (Liang et al., 2017, 2020).

We carry out data preprocessing on all datasets. In the first step, genes with missing values in the samples are deleted. Then, genes contained in both MSKCC and TCGA datasets are selected. Finally, the TCGA samples that exist in MSKCC datasets are removed. After preprocessing, the MSKCC datasets have 4,566 samples and 19,895 genes, which are used as training datasets. The TCGA datasets have 1,262 samples and 19,895 genes as independent datasets. The latter includes 112 BLCA samples, 235 BRCA samples, 170 COADREAD samples, 28 GBM samples, 216 HNSC samples, 41 KIRC samples, 120 LUAD samples, 212 LUSC samples, 22 OV samples, and 106 UCEC samples. For details of all tissue samples of 10 cancers including tumor status and sample sizes after filter, refer to Table 1.

**TABLE 1:** Description of the datasets.

**Primary site**	**Abbreviation**	**Sample numbers of training dataset**	**Sample numbers of independent dataset**
Bladder urothelial carcinoma	BLCA	135	112
Breast invasive carcinoma	BRCA	847	235
Colorectal adenocarcinoma	COADREAD	575	170
Glioblastoma multiforme	GBM	563	28
Head and neck squamous cell carcinoma	HNSC	306	216
Kidney renal clear cell carcinoma	KIRC	490	41
Lung adenocarcinoma	LUAD	356	120
Lung squamous cell carcinoma	LUSC	289	212
Ovarian serous cystadenocarcinoma	OV	562	22
Uterine corpus endometrial carcinoma	UCEC	443	106

### Feature Selection

Each sample contains 19,895 variables whose large number may cause overfitting. As such, we carry out feature selection to improve the generalization ability of classifiers and reduce the time to train the classifier. In the research, Extra tree (Geurts et al., 2006; Geurts and Louppe, 2011) is used for feature selection.

A Wrapper feature selection method based on Extra tree is proposed. Similar idea has been proposed by some researchers utilizing random forest method (Genuer et al., 2010; Verikas et al., 2011). First, variable importance measure is calculated by Extra tree. The variable importance measure of a feature is defined as the average reduction in the classification accuracy after slight random disturbance and before disturbance of the feature out-of-bag (OOB) (Verikas et al., 2011). Then, the sequence backward search is utilized to rank all features according to their importance measures. The feature with the least score is removed from the feature set. The process is iterated until the end of the algorithm. The feature set with the highest classification accuracy and minimum number of variables is selected as the final feature set.

**FIGURE 2:**
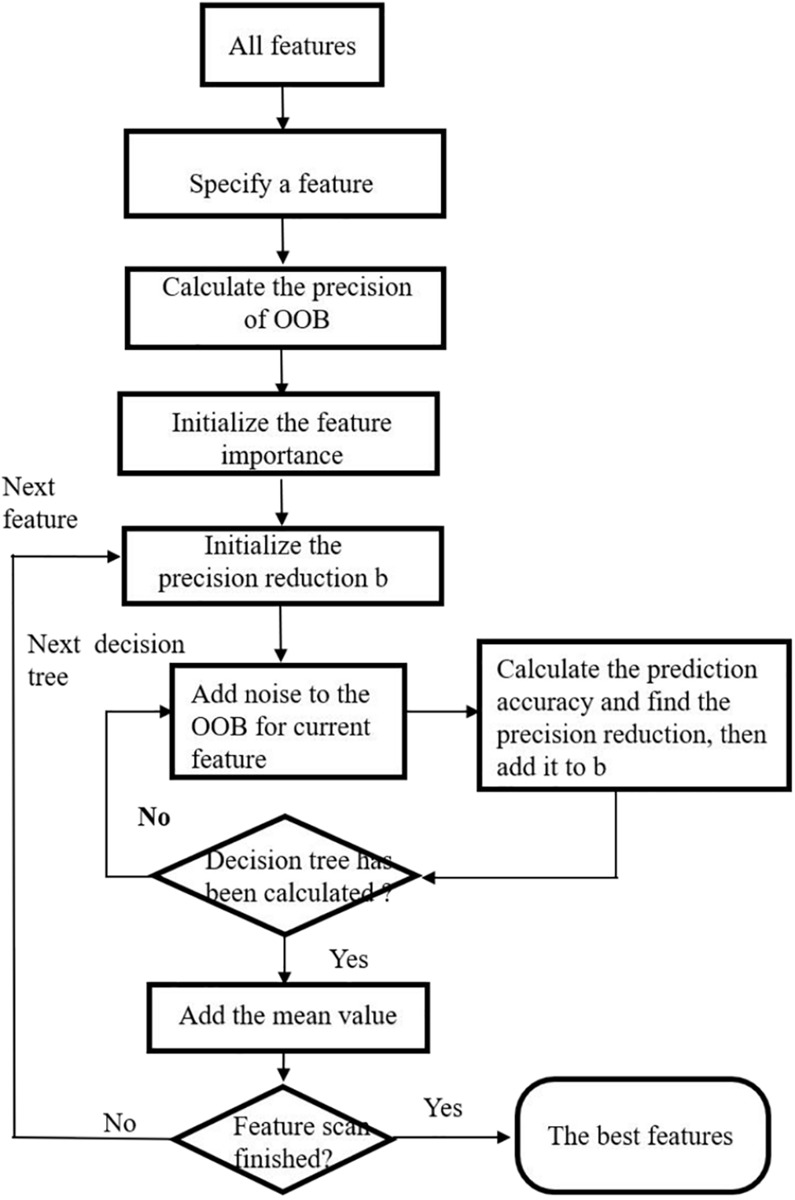
Flow chart of feature selection using extremely randomized tree (Extra tree).

The steps in feature selection using Extra tree are given as follows. Refer to Figure 2 for the flow chart depicting exact process of feature selection.

**Input:** Original dataset with all features and samples.

**Output:** All features with their ranks and corresponding scores from the most important to the least.

**Step 1**. The original dataset is randomly divided into *k* equal parts. We suppose it contained *m* features. *k-1* of them are used to train an Extra tree, and the remaining one is used as test dataset. Therefore, we have *k* extreme random trees *R*_1_,*R*_2_,⋯,*R*_*k*_ in total.

**Step 2**. For each Extra tree, a sequential backward selection method is used to sort the *m* features from the most important to the least. We delete the lowest ranking feature and recorded the average classification accuracy of *R*_*i*_.

**Step 3**. *m* iterations are carried out in **Step 2**, and *R*_*i*_ with the highest classification accuracy is selected as the classification accuracy of the *i*th random tree, and its feature set is obtained.

**Step 4**. For the *k* random trees, the extreme random tree with the highest classification accuracy is used as the final feature selection.

After feature selection, we obtain and rank all features according to their scores from the most important to the least; then, we use principal component analysis (PCA) (Martinez and Kak, 2001; Yang et al., 2004) to perform feature extraction for further analysis.

### XGBoost Classifier

XGBoost classifier is a gradient boosting method incorporating the regression tree (Chen and Guestrin, 2016). It uses the combination of weak learners to create a single strong learner. The idea is to continuously add tree and continuously perform feature splitting to grow a tree.

(1) $$\hat{y_{i}}=\phi \,\left(x_{i}\right)=\sum_{k=1}^{T}f_{k}\,\left(x_{i}\right),f_{k}\in ℱ=,$$

Defining the function as follows.

(2) $$ℱ=\left\{f\left(x\right)=w_{q\,\left(x\right)}\right\}\left(q:R^{m}\to T,w\in R^{T}\right).$$

in which ${\hat{y}}_{i}$ is the predicted label of *i*th sample, *x*_*i*_ represents the *i*th sample, *T* represents the total number of trees, *f*_*k*_ represents the *kth* tree model, and *q* represents the structure of each tree that maps an example to the corresponding leaf index. The objective function of XGBoost classifier is defined as L⁢(ϕ)=∑i=1nl⁢(yi∧i,yi)+∑k=1Ω⁢(fk), where $\Omega \,\left(f\right)=\gamma \,T+\frac{1}{2}\,\lambda \,{||w||}^{2}$. There are two terms in the objective function. The first term is the loss function measuring the difference between the predicted value and the real value. The second term is the regularization term. *T* and *w* refer to the number of leaf nodes and the weight of leaf nodes, respectively. γ controls the number of leaf nodes, and λ is used to prevent overfitting. Each time when a tree is added, it automatically learns a new function to fit the residuals arising from the last prediction. If we obtain *k* trees after training, it is necessary to sum the scores corresponding to each tree to get the predicted value of a sample. In the research, we choose XGBoost classifier, the maximum depth of which is nine, learning rate γ is 0.1, and λ is 0.3 by 10-fold cross-validation as the model for numerical experiments.

## Results and Discussion

### CNVs Performance on Training Datasets

**FIGURE 3:**
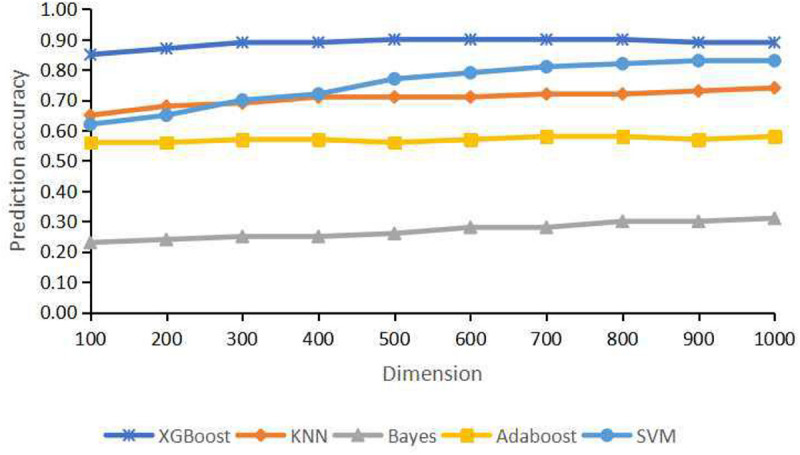
Classification accuracy for different dimensions using extremely randomized tree (Extra tree) and principal component analysis (PCA).

For the training dataset, first, we choose the proper classifier and feature numbers using 10-fold cross-validation with respective overall prediction accuracy. Figure 3 shows the curves of five classifiers changing with dimensions of the training samples. It is found that XGBoost classifier achieves the best result compared with four other classifiers. The overall accuracy changes from 0.8499 to 0.8999 vs. the feature number from 100 to 1,000. The best prediction accuracy is 0.8999 for 700, 800 features of XGBoost classifier. The overall accuracy is 0.8913 using 300 or 400 genes as features for the training datasets. The top 300 features with relatively high between-group variance are more likely to contribute to cancer classification, so we select the top 300 features for training and testing finally.

In this research, the metrics to assess the efficacy of our model included true positives, true negatives, false positives, and false negatives. We also used recall rate, precision, F1 score, and overall prediction accuracy of each cancer for assessing the multiclass predictive performance (Zhao and Zhang, 2020). The recall rate intuitively represents the ability of the classifier to correctly identify all positive cases. Precision is defined as the proportion of the true positives out of all the positive results (both true and false positives). F1 score is the harmonic mean of precision and recall. Accuracy is defined as the ratio (true positive + true negative)/(total number of cases) and calculated for the entire cohort.

**FIGURE 4:**
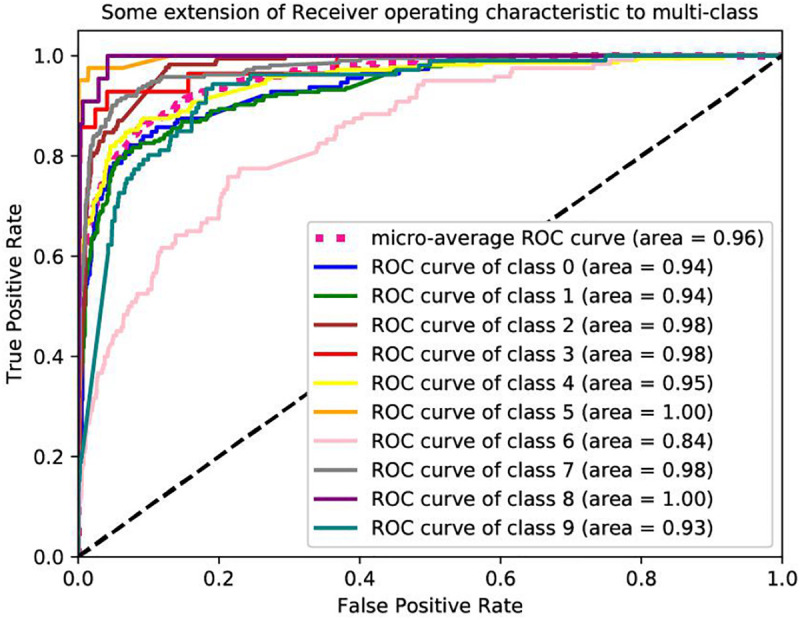
Receiver operating characteristic to predict the tissue of origin of CNV_origin.

Table 2 lists the classification accuracy for each cancer with XGBoost classifier on training datasets via 10-fold cross-validation. Further based on the values of the recall and precision obtained, we plot a receiver operating characteristic curve (ROC) to describe the performance of classification models. The ROCs of these datasets are shown in Figure 4. We are interested in the area under an ROC curve, denoted by AUC, which is another commonly used evaluation criterion. In these experiments, our proposed model achieves fine results. The minimum AUC is 0.84, and microaverage AUC is 0.96.

**TABLE 2:** Classification accuracy for each cancer with XGBoost classifier on training datasets via 10-fold cross-validation.

**Dataset**	**Precision**
BLCA	0.9611
BRCA	0.7852
COADREAD	0.8716
GBM	0.9101
HNSC	0.9378
KIRC	0.9776
LUAD	0.8928
LUSC	0.9453
OV	0.8052
UCEC	0.7988

### Performance Comparison on Independent Datasets

Furthermore, other classifiers, e.g., KNN, SVM, Bayes, and Adaboost, are used to compare with our model with the same benchmark datasets using the same 300 features. In our experiment, we compare the performance of the algorithm to other classical classifiers with the same benchmark datasets. We set the parameters of XGBoost classifier with the learning_rate γ = 0.1, max_depth = 9, andλ = 0.3. The number of categories in KNN is chosen to be equal to five. The parameter n_estimators for Adaboost classifier are 200. For SVM, linear kernel function is chosen with an optional constant *C* = 0.01.

Table 3 gives the performance for independent validation datasets. Clearly, XGBoost classifier performs much better than other classifiers in recall and F1 score on seven cancer datasets including COADREAD, GBM, HNSE, KIRC, LUAD, LUSC, and OV. For other datasets, XGBoost achieves 0.8771 prediction accuracy on BLCA cancer, far higher than that on KNN. For the BRCA dataset, XGBoost achieves an accuracy of 0.7034. For UCEC dataset, XGBoost achieves 0.4864, which is only lower than the Adaboost. For BLCA dataset, the best prediction accuracy comes from XGBoost, with a value of 0.8771.

**TABLE 3:** Comparison with other algorithms on independent datasets.

**Cancer**	**Predictor**	**Precision**	**Recall**	**F1 score**
BLCA	XGBoost	**0.8771**	0.4464	0.5917
	KNN	0.5984	**0.6785**	**0.6359**
	Bayes	0.3232	0.2857	0.3033
	Adaboost	0.8000	0.1785	0.2919
	SVM	0.5555	0.6250	0.5882
BRCA	XGBoost	0.7034	**0.7872**	**0.7429**
	KNN	**0.7777**	0.4170	0.5429
	Bayes	0.3153	0.1489	0.2023
	Adaboost	0.4192	0.6297	0.5034
	SVM	0.7777	0.5361	0.6347
COADREAD	XGBoost	**0.8224**	**0.8176**	**0.8200**
	KNN	0.5265	0.7588	0.6216
	Bayes	0.1250	0.1000	0.1574
	Adaboost	0.6891	0.6000	0.6415
	SVM	0.7986	0.4861	0.7324
GBM	XGBoost	**0.6666**	**0.8571**	**0.7500**
	KNN	0.5135	0.6785	0.5846
	Bayes	0.1250	0.0357	0.0555
	Adaboost	0.5483	0.6071	0.5762
	SVM	0.6176	0.7500	0.6774
HNSE	XGBoost	**0.8369**	**0.7129**	**0.7700**
	KNN	0.6371	0.6667	0.6515
	Bayes	0.3297	0.2870	0.3069
	Adaboost	0.5363	0.5462	0.5412
	SVM	0.6774	0.4861	0.5660
KIRC	XGBoost	**0.7358**	**0.9512**	**0.8297**
	KNN	0.4137	0.8780	0.5625
	Bayes	0.0723	0.9268	0.1342
	Adaboost	0.6341	0.6341	0.6341
	SVM	0.6271	0.9024	0.7400
LUAD	XGBoost	**0.5526**	**0.3500**	**0.4285**
	KNN	0.3535	0.2916	0.3196
	Bayes	0.1212	0.0333	0.0522
	Adaboost	0.2341	0.3083	0.2661
	SVM	0.4869	0.4666	0.4765
LUSC	XGBoost	**0.8310**	**0.8584**	**0.8445**
	KNN	0.7486	0.6745	0.7096
	Bayes	0.5000	0.0849	0.1450
	Adaboost	0.7227	0.3443	0.4664
	SVM	0.7142	0.6603	0.6862
OV	XGBoost	**0.3684**	**0.9545**	**0.5316**
	KNN	0.3090	0.4415	0.7727
	Bayes	0.0408	0.3636	0.0733
	Adaboost	0.2142	0.4090	0.2812
	SVM	0.3508	0.9090	0.5063
UCEC	XGBoost	0.4864	0.6698	0.5669
	KNN	0.4927	0.3207	0.3885
	Bayes	0.0500	0.0094	0.0158
	Adaboost	**0.4965**	**0.7641**	**0.5702**
	SVM	0.3785	0.6792	0.5062

*The bold values in table are the scores of the predictors with the best score in a specific metric for a given type of cancer.*

**FIGURE 5:**
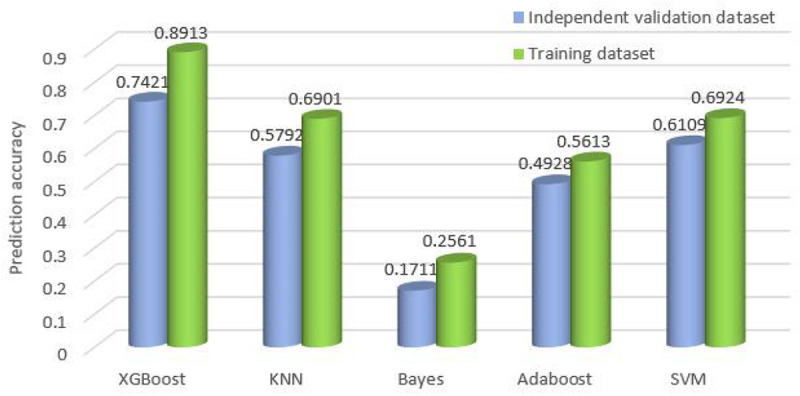
Comparison of the overall accuracy for the classifiers on training datasets and independent validation datasets.

Furthermore, we compare the overall accuracy with the classifiers on all 10 cancer datasets. It is obvious that XGBoost achieves the best results whether on the training datasets or the independent validation datasets. The overall accuracy is 0.7421 and 0.8913, respectively, which is 17 and 22% higher than that of SVM, higher than KNN by 22%, and higher than Adaboost by 37 and 33%. Refer to Figure 5 for the comparison of the overall accuracy between different classifiers based on the training datasets and independent validation datasets.

## Conclusion

In this paper, we propose a cancer type classifier that exploited the copy number variations data of the tumor samples. The copy number variations data contain much noise and is of high dimensionality, so we utilize Extra tree for dimensionality reduction and principal component analysis to reduce noise. Subsequently, XGBoost classifier is applied to 10 types of cancer. Our method achieves the best accuracy regardless of the training datasets or the independent invalidation dataset by selecting 300 features compared with other four classifiers, such as KNN, DNN, SVM, and Adaboost. XGboost classifier adds regular terms to the cost function for controlling the complexity of the model, which contains the number of leaf nodes in the tree and the score sum of squares on each leaf node. From the perspective of bias variance, the regular term reduces the variance of the classifier and simplifies the learned classifier (Chen and Guestrin, 2016), so it can better prevent overfitting in training and its performance is the best compared to the other four classifiers. The results may provide a useful revelation to determine the machine learning model and to understand actual pathological conditions. Furthermore, our results are confirmed by the literatures, with 6 out of top 10 features associated with specific cancers including *NKIRAS1* (Gerashchenko et al., 2010; Khodyrev et al., 2012), *CDKN2B* (Clavell et al., 2008), *YTHDC2* (Wittliff et al., 2015; Yu et al., 2020), *MECOM* (Choi et al., 2017), *RBFOX1* (Sengupta et al., 2013), and *RAB6B* (Kou et al., 2015). The corresponding molecular functions and related cancer of six genes are given in Table 4.

**TABLE 4:** Top six genes and corresponding molecular function.

**ID**	**Gene**	**Molecular function**	**GO annotation**	**Related cancer**
28512	*NKIRAS1*	Intracellular signal transduction	GO:0035556	LUAD KIRC
1030	*CDKN2B*	Positive regulation of transforming growth factor beta receptor signaling pathway	GO:0030511	HNSC
64848	*YTHDC2*	Positive regulation by host of viral genome replication	GO:0044829	COADREAD BRCA
2122	*MECOM*	Negative regulation of JNK cascade	GO:0046329	COADREAD
54715	*RBFOX1*	Regulation of alternative mRNA splicing, via spliceosome	GO:0000381	COADREAD
51560	*RAB6B*	Intra-Golgi vesicle-mediated transport	GO:0006891	COADREAD

*NKIRAS1* regulates the nuclear factor (NF)-kappa B activity by encoding a Ras-like protein. In addition, it is known that the copy number of *NKIRAS1* is usually lower in RCC, and this gene is downregulated in malignant renal tumors.

By forming a complex with *CDK4* or *CDK6*, *CDKN2B* encodes a cyclin-dependent kinase inhibitor. It is found that transforming growth factor (TGF) beta can drastically induce the expression of this gene, suggesting that *CDKN2B* might play roles in TGF-beta-related functions. In addition, the aberrant methylation of p15 (INK4B), a protein encoded by *CDKN2B*, can silence TRβI. Thus, *CDKN2B* and other tumor suppressor genes, such as *CDKN2A*, might be used as biomarkers for early detection in HNSC patient.

*RBFOX1* is a member of the Fox-1 family of RNA-binding proteins, which regulates tissue-specific alternative splicing in metazoa. *RBFOX1* mutations are found in COADREAD cell lines, and the loss of *RBFOX1* may account for the anomalous splicing activity associated with COADREAD.

**FIGURE 6:**
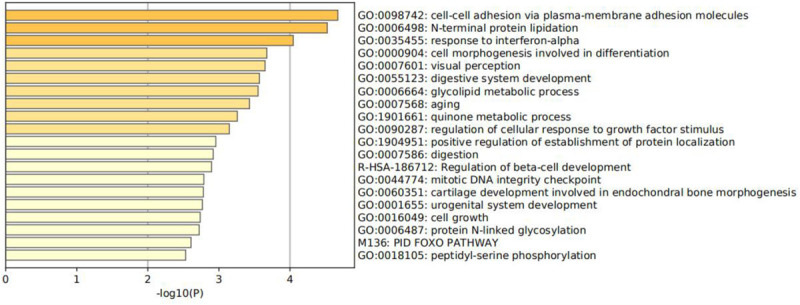
Enriched terms bar graph colored by *p*-values in gene lists.

Function enrichment analysis is performed on the 300 genes. Specifically, all genes have been used as the enrichment background in Figure 6 (Shannon et al., 2003; Zhou et al., 2019). Terms with a *p* <0.01 and an enrichment factor >1.5 are identified and grouped into clusters. Log10(p) is the *p*-value in log base 10. All genes mainly participated in four primary biological pathways to be specific: adhesion via plasma–membrane adhesion molecules (4.61%), cell morphogenesis involved in differentiation (7.45%), positive regulation of establishment of protein localization (4.96%), and cell growth (4.96%).

## Limitations

The traditional clinical pathology experiment has its limitation. Our model recovers the original signal under the condition of oversampling, and the additional signal samples generated by the cancer sample need to be taken into account. Due to the limited data, only 10 types of tumors are selected in this manuscript for the construction of the classifier, and further studies can incorporate more types of tumors into the cancer of unknown primary model in the future work.

## Data Availability Statement

All datasets generated for this study are included in the article/Supplementary Material.

## Author Contributions

YZ conceived the experiments. TF carried out the experiments. SW designed the algorithm. RD analyzed the data. BW contributed reagents, materials, and analysis tools. JY designed the experiments. JS wrote the manuscript. All authors contributed to the article and approved the submitted version.

## Conflict of Interest

BW, RD, and JY were employed by the company Genies (Beijing) Co., Ltd. The remaining authors declare that the research was conducted in the absence of any commercial or financial relationships that could be construed as a potential conflict of interest.

## Footnotes

1 http://cbio.mskcc.org/cancergenomics/pancan_tcga/
